# Caudal Epidural of Pulsed Radiofrequency in Post Herpetic Neuralgia (PHN); Report of Three Cases

**DOI:** 10.5812/aapm.16369

**Published:** 2014-06-25

**Authors:** Olav Jacobus Johannes Maria Rohof

**Affiliations:** 1 Pain Clinic, Orbis Medical Center, Sittard Geleen, The Netherlands

**Keywords:** Chronic Pain, Neuralgia, Postherpetic, Pulsed Radiofrequency Treatment, Anesthesia, Caudal

## Abstract

**Introduction:**

Postherpetic neuralgia (PHN) is a frequently occurring neuropathic pain, its pathophysiology is not fully understood. There are only few evidence based therapeutic options; sympathetic nerve block can be considered for patients with PHN refractory to conservative treatment, but long-term effects are poor. Application of pulsed radiofrequency was effective to treat a variety of pain syndromes without neurological complications or other sequelae.

**Case Presentation:**

We observed a remarkable long-lasting pain relief in patients with post herpetic neuralgia (PHN) treated with caudal epidural PRF. We described the technique of caudal epidural PRF and three case reports.

**Conclusions:**

The mode of action of PRF is far from being completely elucidated. The high frequency current induces an electric field that in turn seems to influence the immunity, the inflammation and other pain conducting mechanisms. Our findings suggest an effect distal from the application of the current. It reaches targets that are difficultly attainable by any other means of current application. The observations of pain relief in the difficult to treat patients with PHN justifies further investigation.

## 1. Introduction

Pulsed radiofrequency (PRF) has been used for the management of a variety of chronic pain syndromes (1, 2). PRF has been used initially as a less neurodestructive alternative for continuous RF (CRF) adjacent to the dorsal root ganglion (3). Its use has been documented in prospective randomized controlled trials (4-7), studying the effect of PRF adjacent to the cervical dorsal root ganglion (DRG), at the Gasserian ganglion, and the medial branch of the lumbar dorsal ramus. PRF adjacent to the DRG proved to be superior to sham and additional CRF does not provide an added value. However, CRF is superior to PRF for the management of facet joint pain and trigeminal neuralgia. Already, a review of the literature obtained information about more than 1200 patients treated for a variety of painful syndromes, including pain induced by peripheral nerves such as the supra scapular nerve. PRF mostly reduced pain without any neurological complications. Since the review, other pain syndromes were treated successfully with PRF. Minimal neurodestructive effects of PRF allows its use for indications that are forbidden for CRF. Intraarticular application of PRF was reported to be successful in a case series (8) and a retrospective study (9). This effect could not be explained by a direct action on neural structures. It was hypothesized that intraarticular PRF has anti-inflammatory effects, potentially induced by an effect on the immune system. Caudal epidural injection of local anesthetics with or without corticosteroids was documented to be effective for the treatment of (sub) acute lumbosacral radicular pain (10). The reported results of intraarticular PRF made us try the caudal route for the management of a patient with severe neuropathic pain after low back surgery refractory to conservative and interventional treatment. An adequate pain relief was noted. Another remarkable result of caudal PRF application with 6 months > 50% pain relief was obtained in a 72-year-old woman with a chronic CRPS, who had undergone a bilateral upper leg amputation 10 years before because of her severe CRPS. Her 35-years-old son, who had > 1.5 years an upper extremity CRPS, not answering to all usual therapies, also requested the same treatment. After the caudal PRF treatment he became pain and complaint free and regained his normal social and professional life. These initial findings made us try to treat a patient with severe post herpetic neuralgia (PHN) and intermittent low back pain. We described three cases where caudal epidural PRF was used for the management of post herpetic neuralgia.

## 2. Case Presentation

All the patients considered for this treatment had received comprehensive conservative and interventional pain treatment modalities. The experimental nature of the caudal epidural PRF treatment was thoroughly discussed and patients were only treated after obtaining a written informed consent.

### 2.1. Assessment and Technique

The patient was asked to indicate all painful areas on a pain drawing. The pain intensity (10-point VAS) and the pain disability index (PDI) were obtained. For the further examination and treatment, the patient lays prone with a pillow positioned under the iliac crests. All spinous processes from the cervical to the sacral segment were examined for tenderness.

**Figure 1.**
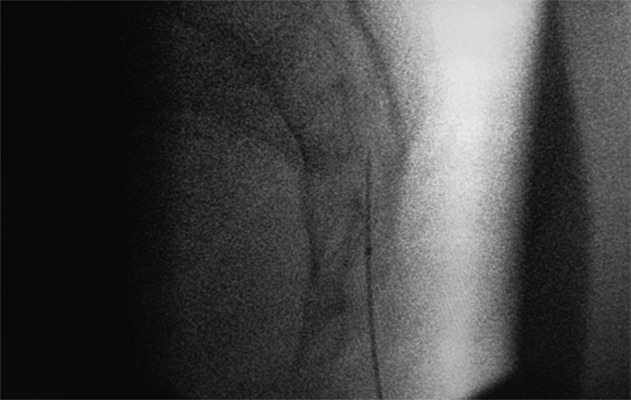
Lateral View: Active Tip Totally in the Caudal Canal

A 20 G Diskit 2 needle (Neurotherm) of 15 cm length with a 20 mm active tip and a radiopaque marker at the end was positioned in the caudal canal under local anesthesia and fluoroscopic lateral view. The 2 cm active tip should lie totally within the distal sacral canal, no further than the S3 level (Figure 1).

**Figure 2.**
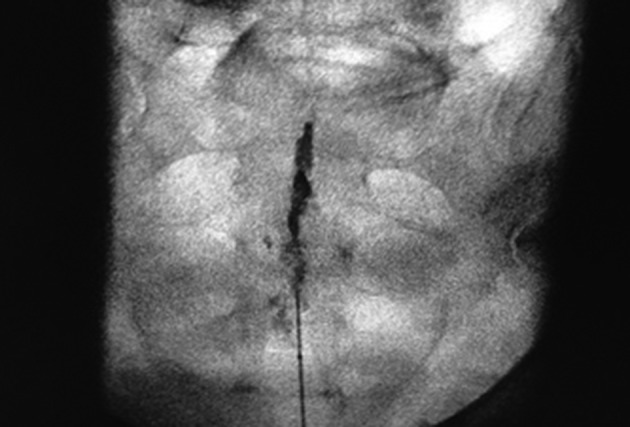
AP view of the epidural space after 0.5mL contrast injection, note the typical “Christmas Tree Picture”

After aspiration to control intraspinal or intravascular needle placement, 1 mL contrast medium (Omnipaque 240 GE HEALTHCARE B. V. Eindhoven, The Netherlands) was injected under fluoroscopic AP view, to confirm epidural position. Most of the contrast medium spreads to the L5 level and the typical “Christmas tree” picture should appear (Figure 2).

The radiofrequency generator for pain management (NT 2000 Neurotherm, Middleton, USA), was connected to the electrode and stimulation at 50 Hz is performed. The ground pad could be applied in the cervical region to cover the vagal nerve.

**Figure 3.**
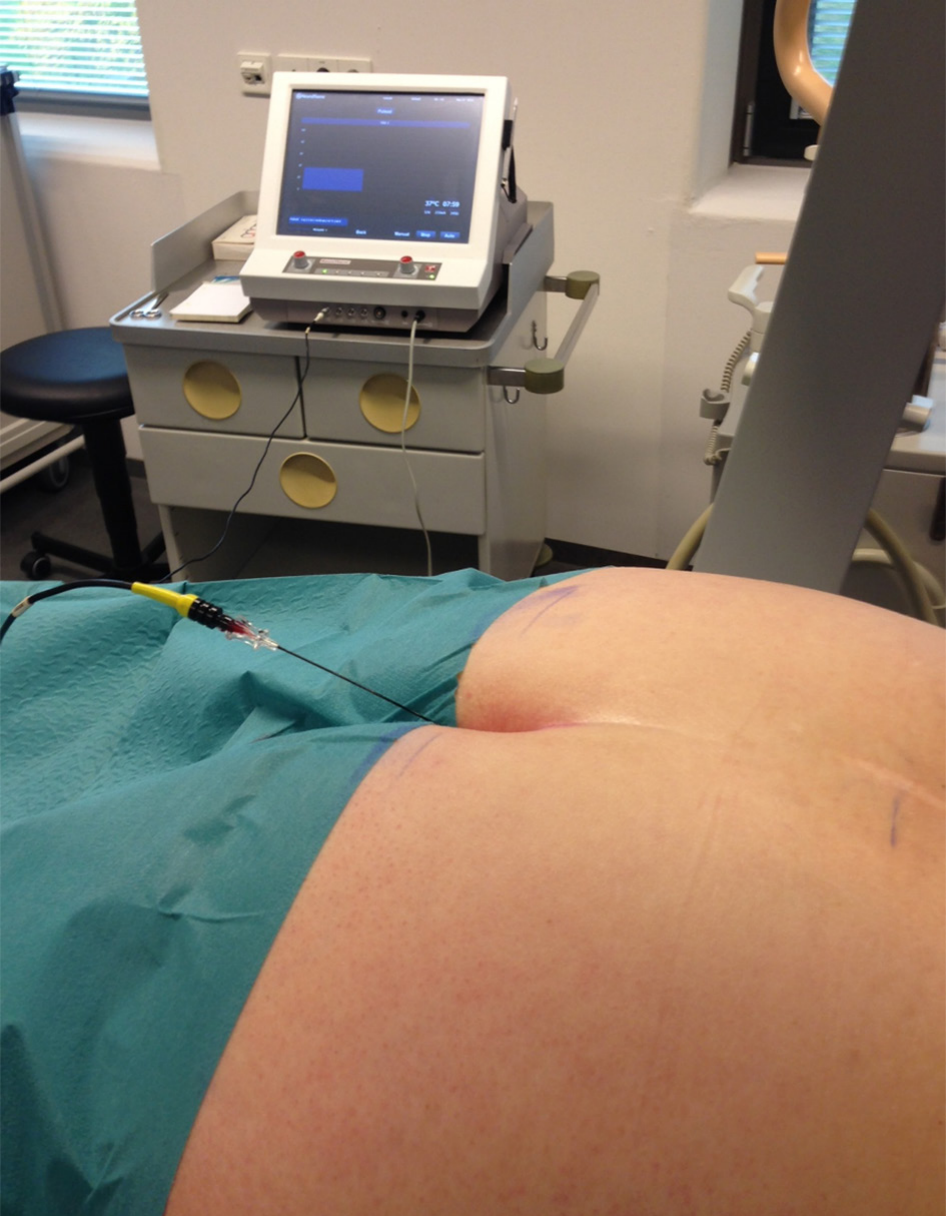
PRF Caudal Application

**Figure 4.**
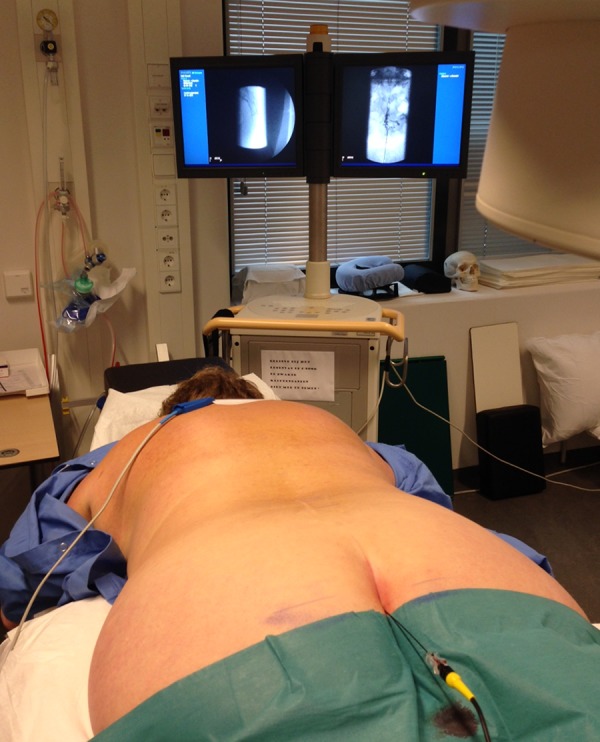
Ground Plate Over Vagal Nerve Neck

Pulsed radiofrequency (PRF) treatment was administered as 5 Hz frequency, 5 ms pulse width, 55 V during 10 minutes and maximum temperature of 42°C. There are several possibilities to use PRF, which was initially 2 Hz 20 ms, meaning that during 1 s 2 pulses of 20 ms duration are given. Later other parameters have been proposed amongst them 5 ms pulse width and a frequency of 5. (Personal communication M. E. Sluijter, the inventor of PRF) the authors use these settings since 2009 in over 4000 cases with good results without significant complications. These PRF parameters were chosen to prevent temperature peaks at the electrode tip and a temperature rise over 42°C with relative high voltages (55 V) in the meantime providing a sufficient powerful electric field to achieve clinical results.

After needle removal, with the patient in prone position, tenderness over the spinous processes and the spontaneous pain level were registered.

The patient was also asked whether any change in pain perception at each painful body area has occurred.

### 2.2. Patient 1. Chronic Low Back Pain and Post Herpetic Neuralgia

A 54-year-old woman was referred with a three-week lasting very painful herpes zoster (HZ) in the dermatomes L1, 2, 3, 4 on the right side. In medical history, intermittent chronic low back pain was found, which usually resolved with conservative therapy. A lumbar epidural corticosteroid and local anesthetic injection relieved pain for only two days. PRF at the DRG L1 and L2 resulted in partial pain relief during 6 weeks. Four months after the initial herpes infection, the patient had PHN with pain, allodynia and spontaneous shooting sensations and low back pain with ischialgia, with no signs of a radicular syndrome.

Caudal epidural PRF treatment (PRF parameters: 39°C, 55 V, 202 mA, impedance 340 Ohm) resulted in immediate pain reduction (7 to 2 on VAS) and improved PDI from 29 to 6. Thirty months after the treatment, the patient was still almost pain free and the back pain and ischialgia were improved.

### 2.3. Patient 2. Post herpetic Neuralgia in a Patient with Insulin Dependent Diabetes Mellitus

A 45-year-old woman, with insulin dependent diabetes mellitus type 2 and hyperthyroidism, had been treated with famciclovir for two weeks and oral pain medication for two months for a left sided herpes zoster Th 12. She had continuous and shooting pain and allodynia indicative for PHN. Epidural corticosteroid injection was considered relatively contraindicated, because of the diabetes. Caudal PRF treatment (PRF parameters: 40°C, 55 V, 184 mA, 285 Ohm) resulted in the typical “stunning” effect. The patient remained totally pain free up till now (10 months).

### 2.4. Patient 3. Post Herpetic Neuralgia in a Patient with Lymphatic Leukemia

A 65-year-old man was admitted with a PHN in the thoracic dermatomes of T11-T12 on the right side, 13 months after an acute HZ. He was known with a chronic lymphatic leukemia treated with 2 mg leukeran per day, which appeared to be in a stable phase. In the acute phase of HZ, he was treated in another hospital with a thoracic epidural corticoid injection, acupuncture, amitriptyline, pregabalin and gabapentin without effect and developed a PHN with chronic pain, allodynia, shooting sensations and itching.

Caudal epidural PRF treatment (PRF parameters: 40°C, 55 V, 194 mA, 246 Ohm) was performed 13 months after the onset of HZ. The patient had no immediate effect and pain was not resolved after 6 weeks of intervention. Acute herpes zoster T11-T12 was relapsed. PRF DRG Th 11 and 12, combined with an epidural corticoid injection had no therapeutic effect.

## 3. Discussion

The initial finding, that caudal PRF application resulted in a period of pain relief in patients with chronic neuropathic pain like a failed back surgery syndrome and two patients with CRPS, showed remarkable longer lasting pain relief in dermatomes far away from the sacral segments. This made us try to treat another group of patients with neuropathic pain, notably patients with chronic refractory PHN. Caudal PRF resulted in immediate pain relief in two of the three patients with PHN.

Only a small percentage of patients with acute HZ infection develop PHN, characterized by excruciating pain, fatigue, diminished mobility and reduced social contacts (11).

The pain from HZ primarily develops because of inflammation of the sensory nerves; the pathophysiology of PHN is not fully understood yet, in any case sensitization and deafferentation play a role in the development of PHN (11, 12). It is our clinical impression that tenderness over almost all spinous processes is a clinical phenomenon pointing at central sensitization. It typically disappears during the caudal PRF, which seems predictive for a longer lasting result.

The immediate stunning effect was mostly lasting one to five days, followed by a flare up of 1-4 weeks in PHN cases, followed by longer lasting pain relief. We speculate that the immediate effect (stunning) could be related to induced changes in neural Sodium Potassium channels, but further studies should confirm this.

Underlying mechanisms of long-term effect of a single caudal epidural PRF application on therapy resistant chronic neuropathic pain are also speculative. We hypothesized that the potential effect may be attributed to the action on the efferent sacral parasympathetic innervation with ganglia in the spinal cord segments of S 2, 3, 4. The afferent parasympathetic information travels with the sympathetic ganglia of S 2, 3, 4 to the spinal cord and the central parasympathetic system, activating the cholinergic anti-inflammatory pathway (13, 14). In an attempt to enhance this effect, the grounding path could be placed over the vagal nerve in the neck, but it is too early to say whether this makes any difference.

In case three, caudal PRF was not effective, which could be caused by relapse of the acute herpes zoster, the leukemia or the time of 13 months elapsed between the acute HZ and PRF caudal treatment. These clinical observations are at least intriguing. Technical parameters to perform caudal epidural PRF were chosen empirically.

High frequency electrical current has been used in a variety of application modes with variable success rates. Our clinical observation that caudal epidural PRF application reduces severe neuropathic pain, refractory to conservative and interventional treatment should be further investigated.
